# Is drinking alcohol associated with sexual coercion among Ugandan university students?: a cross-sectional study

**DOI:** 10.1186/1742-4755-11-7

**Published:** 2014-01-16

**Authors:** Devika Mehra, Anette Agardh, Martin Stafström, Per-Olof Östergren

**Affiliations:** 1 Social Medicine and Global Health, Department of Clinical Sciences, Lund University, Malmö, Sweden; 2 Department of Pediatrics, Centre for Adolescent Health, Royal Children’s Hospital, Murdoch Children’s Research Institute, University of Melbourne, Victoria, Australia; 3 Social Medicine and Global Health, Department of Clinical Sciences, Clinical Research Centre, Lund University, Jan Waldenströms gata 35, House 28, Floor 12, 205 02 Malmö, Sweden

**Keywords:** Sexual coercion, Alcohol, Mental health, Uganda, University students

## Abstract

**Background:**

Sexual coercion is prevalent in sub-Saharan Africa and is a risk factor for unintended pregnancies, sexually transmitted infections and mental health problems. Alcohol consumption patterns have been suggested to be an important factor behind the experience of sexual coercion among university students.

**Objective:**

To study the association between alcohol consumption and the experience of sexual coercion among Ugandan university students.

**Method:**

In 2010, 1954 Ugandan students participated in a cross sectional survey, conducted in Mbarara University of Science and Technology (72% response rate). A self-administered questionnaire assessed socio-demographic factors, alcohol consumption, mental health, and sexual behavior. Multivariable logistic regression analysis was used to examine the various predictors of recent experience of sexual coercion. The data were stratified by sex.

**Results:**

Of the 1954 students, 27.6% reported having experienced sexual coercion and 16.4% stated that they had such an experience recently. Individuals who reported frequent consumption of alcohol, or having consumed alcohol often on the occasion of sexual intercourse, were found to have a higher probability of recent experiences of sexual coercion (OR adjusted 2.29, 95% CI 1.40–3.72, and OR adjusted 2.78, 95% CI 1.56–4.97, respectively). These associations were significant even after adjusting for potential confounders. A synergistic effect was found between poor mental health and frequent consumption of alcohol in conjunction with having sex with regard to its impact on recent experiences of sexual coercion.

**Conclusion:**

We found an association between alcohol consumption and experiences of sexual coercion among Ugandan university students. Therefore, universities may want to consider alcohol prevention under their policy framework, as it could reduce the potential risk of sexual coercion.

## Background

Sexual coercion is a human rights violation and an important public health concern [1]. It has broadly been defined as any situation in which one person uses verbal or physical means including the administration of drugs or alcohol, with or without the other person’s consent to obtain sexual activity [2].

Sexual coercion can have serious physical, psychological and social consequences for the victims [3]. In the short-term these consequences include unintended pregnancies, abortions, and risky sexual behaviors including early onset of consensual sex, multiple sexual partners, and inconsistent condom use [3-5]. The long-term consequences are mostly psychological which may lead to depression, anxiety, suicidal attempts, low self-esteem, low self-efficacy, and substance abuse [3,6].

Most previous research among young people has focused on men as perpetrators and women as victims, but results from studies confirm that men are also victims of sexual coercion perpetrated by men and women [4,7-9]. Several studies in Cameroon, Ghana, and Uganda targeting young people, have found that sexual debut of women was coercive in nature, where men were the perpetrators [5,10-12]. According, to a Ugandan study among adolescents it was found that, 47% of the males report victim experiences and 25% of females report perpetrator behavior [9].

Previous scientific research has found that there is a high prevalence of sexual coercion in Uganda [5,13]. The magnitude of sexual coercion ranges from 5% to 33% among Ugandan university students [13,14]. Most of the research done worldwide on university students shows that sexual coercion occurs among people who know each other, especially in dating partners [15,16]. There could be several reasons for sexual coercion in Uganda, but the most primary reasons are gender power relations that favor men and older age in a relationship [17,18]. The subordinate status of women in Uganda limits their ability to negotiate sexual decisions, which correspondingly increases their risk to unintended pregnancies and other sexually transmitted infections (STI) including HIV, which has a high prevalence in Uganda (7.3%) [19,20].

In Ugandan university students there is a high prevalence of alcohol consumption, a survey done among students at six Ugandan universities showed that “nearly one quarter of all students drink, one fifth of those do so more than five times a month” [14]. Even though, Ugandan universities have a zero tolerance policy against drinking and no alcohol is allowed on campuses, students still continue to drink [21].

Alcohol consumption is a risk factor for sexual coercion among university students [22-24]. It has been seen in prior research that approximately 50% of all cases of sexual coercion involve the use of alcohol or other drugs by the victim or the perpetrator [23]. Alcohol is considered to contribute to the occurrence of rape in Ugandan universities both on and off campus [25]. In a Ugandan context, alcohol consumption may be part of a sexual control mechanism, which is used to obtain sex without the consent of the other person [26]. Sexual coercion can lead to risky sex, as it may not allow the victim to negotiate for safe sex. There are various theories which have been discussed regarding the association between alcohol and sex like alcohol disinhibition [27] and alcohol myopia theory [28]. The latter holds that restriction of cognitive capacity where the person focuses on the salient situational cues of sexual initiation and ignores the peripheral ones, thus limiting their ability to identify the potential dangers, including the risk of sexual coercion. Sex-related alcohol expectancies may influence drinking decisions and subsequently sexual risk-taking behavior among young people [29]. However, these theories have been discussed more in relation to the perpetrators than the victims.

Most of the research on sexual coercion has focused on sexual intercourse and inadequate attention has been given to the continuum of coercion acts, which could be non-penetrative. Lack of data on all types of sexual coercion skews our understanding of sexual coercion.

There is limited research in Uganda that has focused on the drinking patterns of the victims and its association with sexual coercion. Therefore, this study attempts to fill that research gap by investigating the association between alcohol consumption and sexual coercion among Ugandan university students.

## Methods

### Study design and setting

In April 2010, the data for this cross sectional study was collected at the Mbarara University of Science and Technology (MUST), a public university with four faculties: Medicine, Science, Computer Science, and Development Studies. The total university undergraduate student population comprised of 2706 individuals, out of which 1954 participated in the study (72% of all registered undergraduates). The respondents included 1087 males (55.6%) and 867 females (44.4%).

### Data collection and analysis

All students at MUST were invited to participate in the survey. The study questionnaires were distributed to undergraduate students in the lecture halls. They were briefed about the purpose of the study and assured that their responses would remain confidential. Students were also informed that their participation was voluntary and they could withdraw from the study at any time. Consent forms were distributed, signed, and returned anonymously to a sealed box, as were the questionnaires. Contact details of the principal investigator and the research assistant was provided in case they had any queries regarding filling the questionnaire or had any personal concerns. The Institutional Ethics Review Committee at MUST approved the project.

Self-administered survey instrument consisted of an 11 page questionnaire with 132 questions on socio-demographic factors, socio-economic status, role of religion, social capital, alcohol consumption, sexual behavior other lifestyle factors, and mental health. The questionnaire in this study was same as the one used to survey students at MUST in 2005. It was based on validated instruments from other studies [13,30] and on focus group discussions with young people [31].

### Definition of variables

#### Background variables

*Age* was divided into two groups and coded as ≤ 22 (‘younger’) and > 22 (‘older’). The median age of the students was 22 years, which was also used as the cutoff point.

*Area of growing up* was categorised as rural, urban and peri-urban, or small town. The variable was then dichotomised into ‘rural’ or ‘urban’, the latter combining urban, peri-urban and small town.

*Educational level of head of household* had response alternatives did not finish primary school, completed primary school, completed secondary school, post-secondary school, college, university education, and other. This variable was dichotomised as ‘>Primary education’ or ‘≤Primary education’.

*The role of religion in family life while growing up* was dichotomised into ‘major’ (‘religion played a big role or was relatively important’) and ‘minor’ (‘religion was not so important or was not important at all’).

#### Alcohol use

*Alcohol consumption in the past 12 months* was assessed by asking “How often have you consumed alcohol in the last 12 months?” There were five alternative responses: four times per week or more, two to three times per week, three to four times per month, once a month or less, and never. The variable was then trichotomised into ‘abstainers’ (never), ‘infrequent users’ (three to four times per month, once a month or less) and ‘frequent users’ (four times per week or more often, two to three times per week).

*Heavy episodic drinking* (HED) was based on the question “How often do you drink six ‘glasses’ or more on the same occasion?” The following alternative answers could be selected: daily, or almost daily; every week; every month; more seldom than once a month; and never. The variable was then trichotomised into ‘abstainers’ (never), ‘frequent HED’ (daily; or almost daily; every week; every month) and ‘infrequent HED’ (more seldom than once a month and never).

*Alcohol consumption on latest occasion of sexual intercourse* was assessed by two alternatives: ‘yes’ or ‘no’.

*Alcohol consumption on the occasion of sexual intercourse* had the following response alternatives; always, or almost always, more often than on half of the occasions, about half of the occasions, more seldom than on a quarter of the occasions, almost never or never. The first three options were coded as ‘frequent consumers of alcohol on the occasion of sexual intercourse’ and the latter two as ‘infrequent users of alcohol on the occasion of sexual intercourse’.

#### Mental health

The Hopkins Symptom Check List (HSCL-25) was used to assess the mental health status of participants in the survey. This scale consists of 15 items analysing symptoms of depression and 10 items assessing symptoms of anxiety during the week prior to the survey [32]. Furthermore, 10 items from the Symptom Checklist-90 (SCL 90) were included to measure symptoms of psychoticism during the past week prior to the survey. Each item was graded on a four point Likert scale [33]. Both of the above instruments have been validated and applied in different African cultural contexts [13,34,35].

The total mean mental health scores as well as the mean scores of depression, anxiety and psychoticism were calculated from the student’s total score for each measure. Scores were divided by the number of items for all the responses and dichotomised into ‘high score’ and ‘low score’, based on the median spilt between the total scores for each measure. The variables are referred to in the manuscript as ‘poor mental health’ and ‘good mental health’, respectively.

#### Sexual coercion

*Experience of sexual coercion* was measured by means of a validated instrument that has been used in previous studies on sexual behavior [31,36]. Experience of sexual coercion was based on response to the following questions: ‘You have been forced to show your sexual organ’, ‘Someone forced you to let them touch your sexual organ’, ‘Someone forced you to let them suck or lick your sexual organ’, ‘Someone forced you to let them show you their sexual organ’, ‘You have been forced to watch someone masturbate’, ‘You have been forced to masturbate someone’, ‘You have been forced to take part in oral sex or to lick someone’s sexual organ’, ‘You have been forced to take part in sexual intercourse with the penis in the vagina, or someone has inserted an object into your vagina’, or ‘You have been forced to pose for a sex photo or sex film’. If the respondent answered affirmatively to any of these alternatives, they were classified as being exposed to sexual coercion coded as ‘yes’, otherwise they were coded as ‘no’.

*Early experience of sexual coercion was created by combining the variables ‘Age at first time of sexual coercion’ and ‘Current experience of sexual coercion’. We defined all those who reported that* ***their experience of coercion happened before 18 years of age and those who did not report current experience of sexual coercion****, as individuals having had an early experience of sexual coercion.*

#### Dependent variable

*Recent experience of sexual coercion* was based on the response to the questions “How old were you when the coercive experience happened for the first time” and ‘current experience of sexual coercion?’ We classified all those who reported that their first experience of sexual coercion happened at 18 years or above, or were in a situation of current sexual coercion, as exposed to a recent experience of sexual coercion.

### Statistical analysis

The statistical analysis was done using SPSS statistical package Version 20.0. We first calculated the prevalence of all the variables that were used within the sample population. Logistic regression analysis was then carried out to calculate the crude odds ratio (OR) with 95% confidence interval (CI), and determine the associations between the socio-demographic factors, mental health and alcohol consumption patterns (the predictor variables/covariates) regarding recent experience of sexual coercion (the dependent variable).

The multivariable logistic regression analyses was performed to test a set of á priori stated hypotheses, thus the independent variables were chosen beforehand and forced into the model. A step-wise procedure was applied in order to reveal which independent variable, or set of independent variables, that actually made a clear impact on the outcome variable, rather than creating a “black box” by adjusting for all independent variables in one single step*.* Estimates of effect modification were done as “departure from additivity of effects on the chosen outcome scale”. In addition, a synergy index (SI) was calculated [37] to disclose effect modification between the chosen variables, as proposed by Rothman [38].

## Results

Table 1 gives the prevalence of all the socio-demographic factors, alcohol consumption, mental health, and experience of sexual coercion in our sample of Ugandan university students.

**Table 1:** Prevalence of socio-demographic factors, alcohol consumption, mental health, and sexual coercion among Ugandan university students

		**All**	**Male**	**Female**	**χ**^**2**^
		**n (%)**	**n (%)**	**n (%)**	**p**^**1**^
		**1954 (%)**	**1087 (%)**	**867 (%)**	
Age	≤22	1042 (55.5)	521 (49.6)	521 (63.1)	< .001
	>22	835 (44.5)	530 (50.4)	305 (36.9)	
	Missing	77	36	41	
Area of growing up	Urban	1067 (55.1)	551 (51.2)	516 (60.1)	< .001
	Rural	869 (44.9)	526 (48.8)	343 (39.9)	
	Missing	18	10	8	
Educational level of head of the household	>Primary education	1382 (72.7)	730 (68.9)	652 (77.5)	< .001
	≤Primary education	518 (27.3)	329 (31.1)	189 (22.5)	
	Missing	54	28	26	
Importance of religion	Major	1232 (63.5)	650 (60.3)	582 (67.5)	.001
	Minor	708 (36.5)	428 (39.7)	280 (32.5)	
	Missing	14	9	5	
Mental health	Good mental health	920 (50.4)	549 (54.5)	371 (45.5)	< .001
	Poor mental health	904 (49.6)	459 (45.5)	445 (54.5)	
	Missing	130	79	51	
Experience of sexual coercion	No	1130 (72.4)	665 (77.5)	456 (66.1)	< .001
	Yes	431 (27.6)	193 (22.5)	238 (33.9)	
	Missing	393	229	164	
Early experience of sexual coercion^2^	No	1386 (88.8)	766 (89.3)	620 (88.2)	.519
	Yes	175 (11.2)	92 (10.7)	83 (11.8)	
	Missing	393	229	164	
Recent experience of sexual coercion^2^	No	1305 (83.6)	757 (88.2)	548 (78.0)	< .001
	Yes	256 (16.4)	101 (11.8)	155 (22.0)	
	Missing	393	229	164	
Alcohol consumed in the past 12 months	Abstainers	1017 (56.2)	514 (49.9)	503 (64.5)	< .001
	Infrequent users	632 (34.9)	403 (39.1)	229 (29.4)	
	Frequent users	161 (8.9)	113 (11.0)	48 (6.2)	
	Missing	144	57	87	
Heavy episodic drinking (HED)	Abstainers	1017 (58.0)	514 (51.9)	503 (66.1)	< .001
	Seldom HED	530 (30.3)	326 (32.9)	204 (26.8)	
	Frequent HED	205 (11.7)	151 (15.2)	54 (7.1)	
	Missing	202	96	106	
Alcohol on latest occasion of sexual intercourse^3,4^	No	360 (72.9)	227 (70.7)	133 (76.9)	.168
	Yes	134 (27.1)	94 (29.3)	40 (23.1)	
	Missing	96	67	29	
Alcohol consumption on the occasion of sexual intercourse^3,4^	Seldom users	391 (79.0)	245 (76.6)	146 (83.4)	.084
	Frequent users	104 (21.0)	75 (23.4)	29 (16.6)	
	Missing	95	68	27	

^1^p value analysed in the table is based on sex.

^2^Only analyzed for those individuals who had experienced sexual coercion.

^3^Only analyzed for those who have consumed alcohol.

^4^Only analyzed for individuals who had had sexual intercourse.

The age of the participants was dichotomized into ≤22 and >22 (55.5% and 44.5% respectively). The participants who came from the urban background totaled 55.1% and from the rural area were 44.9%. Approximately, 72.7% of the respondent’s grew up in an environment where the educational level of head of the household had more than primary education and 27.3% had less than primary education. Approximately 28% of our sample had an experience of sexual coercion. We observed some gender differences, a larger proportion of females (33.9%) reported such coercion compared to males (22.5%). Approximately one-sixth of the sample had a *recent* experience of sexual coercion with a higher prevalence in females (22%) compared to males (11.8%). More than half of the students surveyed abstained from using alcohol in the past 12 months and less than one-tenth were frequent consumers of alcohol. The proportion of students consuming alcohol on the occasion of sexual intercourse was higher for seldom users (79%) than frequent users (21%).

Table 2 shows the bivariate associations between socio-demographic factors, alcohol consumption, and mental health regarding recent experience of sexual coercion. We observed significant gender differences in the findings. Male students over 23 years and who came from families where the educational level of the head of household was less than primary school had higher odds of having a recent experience of sexual coercion. Students with poor mental health were three times more likely to report sexual coercion, a finding that was significant for males and females. Alcohol consumption over the past 12 months had a significant association with recent experience of sexual coercion (OR crude 2.14, 95% CI 1.38–3.32), the finding was significant for both males (OR crude 2.21, 95% CI 1.07–4.55) and for females (OR crude 2.21, 95% CI 1.07–4.55). The association between alcohol consumption on the occasion of sexual intercourse was significant with recent experiences of sexual coercion (OR crude 3.14, 95% CI 1.84–5.37) and showed considerable gender differences. Females had a higher risk (OR crude 5.65, 95% CI 2.24–14.22) compared to males (OR crude 2.53, 95% CI 1.27–5.05).

**Table 2:** Association (Crude Odds ratios, 95% Confidence Interval) between socio-demographic variables, mental health, and alcohol consumption patterns with recent experience of sexual coercion among Ugandan university students

		**All**		**Male**		**Female**	
		**n (%)**	**OR (95% CI)**	**n (%)**	**OR (95% CI)**	**n (%)**	**OR (95% CI)**
Age	≤ *22*	124 (14.6)	1 (Ref)	34 (8.1)	1 (Ref)	90 (20.7)	1 (Ref)
	>22	124 (19.0)	1.37 (1.04–1.80)	64 (15.6)	2.08 (1.34–3.23)	60 (24.8)	1.26 (.86–1.82)
Area of growing up	Urban	126 (14.8)	1 (Ref)	43 (9.9)	1 (Ref)	83 (20.0)	1 (Ref)
	Rural	127 (18.2)	1.28 (.97–1.67)	56 (13.5)	1.41 (.93–2.16)	71 (25.2)	1.35 (.94–1.93)
Educational level of head of the household	> Primary education	170 (15.4)	1 (Ref)	59 (10.2)	1 (Ref)	111 (21.2)	1 (Ref)
	≤ Primary education	82 (19.3)	1.31 (.98–-1.75)	42 (16.0)	1.67 (1.09-2.55)	40 (24.8)	1.22 (.81-1.85)
Importance of religion	Major	153 (15.8)	1 (Ref)	58 (11.6)	1 (Ref)	95 (20.4)	1 (Ref)
	Minor	101 (17.2)	1.10 (.84–1.45)	42 (12.0)	1.04 (.68–1.59)	59 (24.9)	1.29 (.89–1.87)
Mental health	Good mental health	68 (8.9)	1 (Ref)	30 (6.7)	1 (Ref)	38 (12.1)	1 (Ref)
	Poor mental health	180 (23.9)	3.21 (2.38–4.33)	66 (17.1)	2.90 (1.84–4.57)	114 (30.9)	3.24 (2.16–4.86)
Alcohol consumption in the past 12 months	Abstainers	125 (14.8)	1 (Ref)	40 (9.4)	1 (Ref)	85 (20.3)	1 (Ref)
	Infrequent users	78 (15.5)	1.05 (.77–1.43)	38 (12.1)	1.04 (.68–1.59)	40 (21.1)	1.33 (.83–2.12)
	Frequent users	34 (27.2)	2.14 (1.38–3.32)	21 (23.6)	2.21 (1.07–4.55)	13 (36.1)	2.97 (1.65–-5.35)
Alcohol consumption on the occasion of sexual intercourse^1,2^	Infrequent users	54 (16.5)	1 (Ref)	29 (14.4)	1 (Ref)	25 (19.8)	1 (Ref)
	Frequent users	31 (38.3)	3.14 (1.84–5.37)	17 (29.8)	2.53 (1.27–5.05)	14 (58.3)	5.65 (2.24–14.22)

^1^Only analyzed among individuals who had had sexual intercourse.

^2^Only analyzed among individuals who had consumed alcohol.

Table 3 shows the multivariate analysis regarding the association between alcohol consumption in the last 12 months with recent experience of sexual coercion, which was found to be significant in the final model (OR adjusted 2.29, 95% CI 1.40–3.72). The multivariate analysis indicated that there was possible confounding between alcohol consumption and poor mental health. It was also found that females older than 23 years were at a greater risk for being sexually coerced. Table 4 shows the multivariate association between alcohol consumption on the occasion of sexual intercourse and recent experiences of sexual coercion The association remained significant even after adjusting for confounders (OR adjusted 1.79, 95% CI 1.05–.04). The analysis showed that that there was possible confounding between alcohol consumption and poor mental health and females were found to be at a higher risk of being recently sexually coerced.

**Table 3:** Association (Adjusted Odds Ratio, 95% Confidence Interval) between alcohol consumption in the past 12 months and recent experience of sexual coercion among Ugandan university students

	**Model 1**	**Model 2**^**1**^	**Model 3**^**2**^	**Model 4**^**3**^	**Model 5**^**4**^	**Model 6**^**5**^
	**OR (95% CI)**	**OR (95% CI)**	**OR (95% CI)**	**OR (95% CI)**	**OR (95% CI)**	**OR (95% CI)**
Alcohol consumption in the last 12 months						
Abstainers	1 (Ref)	1 (Ref)	1 (Ref)	1 (Ref)	1 (Ref)	1 (Ref)
Infrequent users	.98 (.71–1.36)	1.09 (.79–1.52)	1.09 (.79–1.52)	1.11 (.80–1.55)	1.12 (.80–1.56)	1.02 (.72–1.43)
Frequent users	2.12 (1.34–3.33)	2.62 (1.64–4.18)	2.61 (1.63–4.17)	2.68 (1.67–4.29)	2.73 (1.70–4.39)	2.29 (1.40–3.72)
Female		2.30 (1.70–3.11)	2.48 (1.82–3.38)	2.53 (1.85–3.45)	2.57 (1.88–3.51)	2.37 (1.72–3.25)
>22 years			1.62 (1.20–2.19)	1.56 (1.15–2.11)	1.53 (1.13–2.08)	1.64 (1.20–2.23)
Rural				1.30 (.96–1.76)	1.21 (.88–1.67)	1.17 (.84–1.62)
Low educational level of head of household					1.25 (.88–1.76)	1.08 (.76–1.54)
Poor mental health						3.14 (2.25–4.37)

^1^Model 2 adjusted for sex.

^2^Model 3 adjusted for sex and age.

^3^Model 4 adjusted for sex, age, and area of origin.

^4^Model 5 adjusted for sex, age, and area of origin and educational level of head of household.

^5^Model 6 adjusted for sex, age, and area of origin and educational level of head of household, and mental health.

**Table 4:** Association (adjusted odds ratio, 95% confidence interval) between alcohol consumption on the occasion of sexual intercourse and recent experience of sexual coercion among Ugandan university students

	**Model 1**	**Model 2**^**1**^	**Model 3**^**2**^	**Model 4**^**3**^	**Model 5**^**4**^	**Model 6**^**5**^
	**OR (95% CI)**	**OR (95% CI)**	**OR (95% CI)**	**OR (95% CI)**	**OR (95% CI)**	**OR (95% CI)**
Frequency of alcohol on the occasion of sexual intercourse						
Infrequent users	1 (Ref)	1 (Ref)	1 (Ref)	1 (Ref)	1 (Ref)	1 (Ref)
Frequent users	2.95 (1.7–5.12)	3.18 (1.82–5.57)	3.14 (1.79–5.51)	3.17 (1.81–5.57)	3.21 (1.82–5.65)	2.78 (1.56–4.97)
Female		1.88 (1.14–3.10)	1.97 (1.18–3.29)	2.01 (1.20–3.37)	2.03 (1.21–3.40)	1.79 (1.05–3.04)
>22 years			1.36 (.82–2.25)	1.34 (.81–2.23)	1.31 (.79–2.19)	1.43 (.85–2.42)
Rural				1.14 (.68–1.90)	1.09 (.64–1.86)	.95 (.55–1.65)
Low educational level of head of household					1.19 (.65–2.18)	1.14 (.61–2.11)
Poor mental health						2.72 (1.52–4.85)

^1^Model 2 adjusted for sex.

^2^Model 3 adjusted for sex and age.

^3^Model 4 adjusted for sex, age, and area of origin.

^4^Model 5 adjusted for sex, age, and area of origin and educational level of head of household.

^5^Model 6 adjusted for sex, age, and area of origin and educational level of head of household, and mental health.

To further investigate the association between alcohol consumption and recent experience of sexual coercion, we analysed whether mental health was an effect modifier (Tables 5 and 6) and found that frequent alcohol use on the occasion of sexual intercourse and poor mental health had a synergistic effect on recent experience of sexual coercion (Table 6).

**Table 5:** Analysis of effect modification between alcohol consumption in the last 12 months and mental health regarding recent experience of sexual coercion in a sample of Ugandan university students (n = 1954), presented as adjusted odds ratios (OR) with 95% confidence intervals (CI)

	**n (%)**	**Cases**	**OR (95% CI)**	
Infrequent user of alcohol and good mental health	811 (47.5)	52	1 (Ref)	
Infrequent user of alcohol and poor mental health	744 (43.5)	145	3.57 (2.54–5.01)	
Frequent user of alcohol and good mental health	60 (3.5)	10	3.24 (1.52–6.89)	
Frequent user of alcohol and poor mental health	94 (5.5)	23	5.20 (2.95–9.16)	Synergy index = 0.87
Missing	245			

**Table 6:** Analysis of effect modification between alcohol consumption on the occasion of having sexual intercourse and mental health regarding recent experience of sexual coercion in a sample of Ugandan university students (n = 1954), presented as adjusted Odds Ratios (OR) with 95% Confidence Intervals (CI)

	**n (%)**	**Cases**	**OR (95% CI)**	
Infrequent user of alcohol on the occasion of sexual intercourse and good mental health	176 (37.3)	13	1 (Ref)	
Infrequent of alcohol on the occasion of sexual intercourse and poor mental health	197 (41.7)	40	3.05 (1.56–5.96)	
Frequent user of alcohol on the occasion of sexual intercourse and good mental health	31 (6.6)	5	2.57 (.83–8.00)	
Frequent user of alcohol on occasion of sexual intercourse and poor mental health	68 (14.4)	24	8.24 (3.77–18.03)	Synergy index = 2.00
Missing	118			

## Discussion

In this study among Ugandan university students, we found that those students, who frequently consumed alcohol or used it in relation to sex, had a higher risk of being subjected to sexual coercion. These associations were statistically significant even after adjusting for potential confounders. We also found a synergistic effect between frequent consumers of alcohol on the occasion of sexual intercourse and poor mental health with recent experience of sexual coercion. But, no synergy was found in the association between alcohol consumption in general during the past 12 months and poor mental health in its bearing on recent experience of sexual coercion.

From the current study we found that alcohol consumption in the last 12 months had an association with recent experience of sexual coercion. A possible explanation for this could be that in a university environment, where students socialize at bars or parties, risky alcohol consumption may lead to sexual coercion. A previous study suggests that there is a causal link between alcohol consumption at bars or parties, and the occurrence of sexual coercion [39]. It would be difficult to disentangle the direct effect of the alcohol consumed by the victim, from the effects associated with the setting, where the perpetrators are present [39].

We also found an association between alcohol consumption in relation to sex with recent experience of sexual coercion. This association can be explained by various mechanisms. At a behavioral or psychological level, alcohol may decrease the risk perception and the ability to communicate assertively, making an individual more vulnerable to being sexually victimized [23,39,40]. There are various theories that have been discussed in literature regarding the association between alcohol and sex, like sexual disinhibition or alcohol myopia theory [41,42] which explains that due to the high consumption of alcohol the person might not be able to identify the potential dangers, including the risk of being sexually coerced.

However, even if these theories explain some behavior, the perpetrators are responsible for the act of sexual coercion, regardless of whether they were intoxicated or not [23]. In a coercive situation the victim is left without any decision making latitude which can lead to unsafe sex that can further result in unintended pregnancy or an STI. Studies have shown that women who have consumed alcohol were more likely to report that they would respond passively to unwanted sexual advances [43] and would be able to employ less effective refusal strategies when they face sexual aggression than sober women [23]. Therefore, it is important to empower women themselves, by identifying actions that may put them at a risk.

Alcohol consumption may also act as a coping mechanism to reduce psychological distress among victims, which may also be lead to abuse over time [44]. In our analyses, poor mental health confounded the association between alcohol use and sexual coercion. Still, alcohol use indicators remained statistically significant, suggesting that alcohol consumption and poor mental health represent two independent causal mechanisms. In addition, we also found a synergistic effect between frequent alcohol consumption on the occasion of sexual intercourse and poor mental health, regarding the risk for recent experience of sexual coercion.

Among gender differences, females who consumed alcohol were at a higher risk of being sexually coerced compared to males. There is a discrepancy in the existing gender norms regarding drinking patterns in Uganda. Consuming alcohol is related to masculinity among young boys and is associated with lack of respect among girls [45]. The cultural gender stereotypes consider women who drink to be sexually available and more likely to engage in sexual intercourse than women who abstain [39,46]. These stereotypes are accentuated by gender imbalances in Uganda, men have the power to control women’s behavior, including the sexual decision making process. There is evidence from previous research that alcohol consumption facilitates coercive acts because of the alcohol myopia mechanism, which render them less sensitive to the victim’s signs of refusal, which can accentuate the likelihood of sexual or physical violence [23,26].

### Strengths and limitations

The survey, on which our study was based, targeted all students at MUST, with a total response rate of 72%, which leaves room for selection bias. However, the reasons for non-participation do not seem to be linked to the main exposures nor to the outcome, since they could be due to many reasons, which might include logistic circumstances, length of the questionnaire, or unwillingness to participate due to the sensitive questions in the survey. But, we do not have any data to support those assumptions. Strength of our study was that we focused on the drinking patterns of victims and their association with recent experience of sexual coercion. A limitation of our study stems from its cross-sectional design, which does not permit us to safely judge the direction of causation between the independent variables and the self-reported experiences of sexual coercion. A reverse causation could not be ruled out; on the contrary it seems plausible, but we find it unlikely to be the major mechanism behind our findings. Another potential limitation of our study was that there was no information in the questionnaire on the drinking patterns of the perpetrators of sexual coercion. The prevalence of students who were exposed to sexual coercion and alcohol consumption may have been higher than reported in the questionnaire, since socially undesirable behaviors are usually underreported. However, we believe that if this were so, it would bias the differences we found towards the null, since it would more likely represent a case of non-differential misclassification than one of differential misclassification. In our analyses we adjusted for the most obvious confounding factors, including those that had little impact on the associations that we determined.

## Conclusion

This study concludes that there is an association between alcohol consumption patterns and sexual coercion among Ugandan university students**.** Universities should consider alcohol use prevention under their policy framework, as it could reduce the potential risk of sexual coercion, in addition to other harmful effects of alcohol abuse. It is crucial for universities to develop institutional capacity for handling cases of sexual coercion. One consideration can be to conduct training workshops that involve both men and women, in order to create awareness regarding the existing gender inequalities, and the importance of respecting each other’s sexual rights. These trainings can be used to inform on the process of reporting cases of coercion and the consequences for the perpetrators. This can help in reducing negative reproductive health outcomes such as teenage pregnancy and sexually transmitted infections. For future research we would like to suggest that it might be beneficial to conduct longitudinal research to confirm the association between alcohol consumption and sexual coercion.

## Competing interests

The authors declare that they have no competing interests.

## Authors’ contributions

DM contributed to the data collection, study design, conducted the statistical analysis, analysed the data and drafted the manuscript. PO contributed to the study design, data analysis, interpretation of results and in the writing of the manuscript. AA designed the study and developed the protocol, collected the data, contributed to the data analysis and in writing of the manuscript. MS contributed to the data analysis and in the writing of the manuscript. All authors have read and approved the final manuscript.
